# A biopsychosocial interpretation of the Neuropsychiatric Inventory – Nursing Home assessment: reconceptualising psychiatric symptom attributions

**DOI:** 10.1192/bjo.2020.113

**Published:** 2020-11-06

**Authors:** Sarah J. Smith, Alys W. Griffiths, Byron Creese, Cara Sass, Claire A. Surr

**Affiliations:** Centre for Dementia Research, Leeds Beckett University, UK; Centre for Dementia Research, Leeds Beckett University, UK; College of Medicine and Health, University of Exeter Medical School, UK; Centre for Dementia Research, Leeds Beckett University, UK; Centre for Dementia Research, Leeds Beckett University, UK

**Keywords:** Psychiatric nursing, psychological testing, rating scales, dementia, carers

## Abstract

**Background:**

The Neuropsychiatric Inventory (NPI) is predicated on the assumption that psychiatric symptoms are manifestations of disease. Biopsychosocial theories suggest behavioural changes viewed as psychiatric may also arise as a result of external behavioural triggers. Knowing the causes of psychiatric symptoms is important since the treatment and management of symptoms relies on this understanding.

**Aims:**

This study sought to understand the causes of psychiatric symptoms recorded in care home settings by investigating qualitatively described symptoms in Neuropsychiatric Inventory-Nursing Home (NPI-NH) interviews.

**Method:**

The current study examined the NPI-NH interviews of 725 participants across 50 care homes. The qualitatively described symptoms from each of the 12 subscales of the NPI were extracted: 347 interviews included at least one qualitatively described symptom (*n* = 651 descriptions). A biopsychosocial algorithm developed following a process of independent researcher coding (*n* = 3) was applied to the symptom descriptions. This determined whether the description had predominantly psychiatric features, or features that were cognitive or attributable to other causes (i.e. issues with orientation and memory; expressions of need; poor care and communication; or understandable reactions)

**Results:**

Our findings suggest that the majority (over 80%) of descriptions described symptoms with features that could be attributable to cognitive changes and external triggers (such as poor care and communication).

**Conclusions:**

The finding suggest that in its current form the NPI-NH may over attribute the incidence of psychiatric symptoms in care homes by overlooking triggers for behavioural changes. Measures of psychiatric symptoms should determine the causes of behavioural changes in order to guide treatments more effectively.

## Background

Psychiatric symptoms are common in dementia and include disturbances of mood, perception and behaviour such as depression, apathy, disinhibition and hallucinations.^1^ The prevalence of psychiatric symptoms in care home residents ranges from 40 to 85%^2^ representing a challenge for care providers and policymakers. Although treatment includes pharmacological and non-pharmacological options, prescribing antipsychotics to manage psychiatric symptoms is contentious because of limited efficacy and long-lasting side-effects.^3^ Providing and developing appropriate and effective treatments relies on the accurate identification of psychiatric symptoms as they occur.

## The Neuropsychiatric Inventory

The Neuropsychiatric Inventory (NPI)^4^ represents a well-established measure of psychiatric symptoms in dementia frequently used in randomised controlled trials of pharmacological and non-pharmacological interventions.^5,6^ The Neuropsychiatric Inventory – Nursing Home (NPI-NH) version^7^ is a proxy interview-based measure, predicated on ten behavioural and two neurovegetative categories.

However, there are noted limitations of the NPI (and subsequent NPI-NH). Namely that they are predicated on the assumption that psychiatric symptoms are manifestations of disease,^4^ and not designed to distinguish between behaviours caused by disease and behaviours that represent a reaction to the physical or social environment.^5^

## Classification of symptoms: psychiatric versus neurological

Since the NPI was developed the extent to which pathology contributes to psychiatric symptoms, and the degree to which neurological and psychiatric symptoms overlap in dementia, has been debated. For example, Crossley et al^8^ sought to determine, by meta-analysis of neuroimaging evidence, whether distinct brain regions are implicated in psychiatric and neurological symptoms; comparing the brain regions that had been implicated in 24 psychiatric and neurological conditions (as described in the ICD-10), drawing on data from at least seven voxel-based morphometry studies for each disorder. The disorders included several types of dementia and psychiatric disorders. Their findings implicated distinct regions in psychiatric (cingulate, medial frontal, superior frontal and occipital cortex) versus neurological (basal ganglia, insula, sensorimotor and temporal cortex) disorders. In their initial analysis dementia was classified as a neurological disorder, although dementias are described as both neurological and psychiatric in the ICD-10. Confirmatory analysis in which the dementias (Alzheimer's disease, frontotemporal and dementia in Parkinson's disease) were classified as psychiatric disorders was also conducted. In this subsequent confirmatory analysis, classifying dementias as psychiatric disorders led to changes in the degree to which temporal regions were associated with psychiatric disorders. The temporal cortex was primarily implicated in neurological disorders when dementias were classified as neurological, whereas it was primarily implicated in psychiatric disorders when dementias were classified as psychiatric.

These findings speak to the difficulty of classifying psychiatric symptoms in dementia. Dementia is primarily considered a neurological disorder associated with cognitive symptomology, with the tendency for psychiatric symptoms to manifest in later stages.^9^ Only in less common types of dementia are psychiatric symptoms a hallmark of the dementia phenotype for example frontal dementia and dementia with Lewy bodies.^10^ The findings from Crossley et al that indicate that brain regions associated with cognitive symptoms are implicated when dementia is treated as a psychiatric illness may suggest that cognitive changes drive psychiatric symptoms in dementia.^8^

This view is consistent with a biopsychosocial approach, in which psychiatric symptoms can be understood as arising from the interplay between neurological changes expressed as cognitive symptoms and environmental triggers, or as the result of understandable reactions to care being provided. If this is the case then symptoms may be amenable to treatment by manipulating or changing the environment or caregiving interactions.

## Biopsychosocial approach

A range of external factors may cause expression of psychiatric symptoms in dementia, such as unmet needs and lack of activity,^11^ environmental triggers^12^ and the interactions between people with dementia and their caregivers.^13^ A biopsychosocial approach can be applied to understand the degree to which behavioural changes are a function of the interaction between the person (including neurobiological changes and cognitive symptoms), their personal history and personality, and the social environment in which they exist.^14^

The NPI-NH in its current form endorses reporting behaviours as part of a unified neuropsychiatric symptomology regardless of the degree to which the symptom is predicated on cognitive, psychiatric or external triggers. For example, one of the questions related to symptoms of agitation is ‘Does the resident get upset when people are trying to care for him/her or resist activities such as bathing or changing clothes?’ in the context of the NPI a person experiencing reluctance and distress when entering a bathroom would be unilaterally labelled as agitated. Applying the principles of a biopsychosocial approach the same behaviours may represent an understandable reaction to the distress caused by not understanding why they are entering a bathroom (cognitive changes) and having personal clothing removed by a stranger (external cues).

Recent studies using the NPI have identified that levels of psychiatric symptoms vary across settings suggesting that the NPI is picking up on environmental cues, even though this is not being recorded.^15^ For example, lower levels of apathy are observed in services where there are more staff-led activities for residents. This indicates that although the NPI does not seek to distinguish between environmentally triggered behaviours it is sensitive to environmental and social triggers.

## Aims

In the current study we sought to explore the types of behaviours described as psychiatric symptoms in the NPI-NH, adopting an approach similar to previous research in clinical settings that used algorithms to distinguish between neurological (cognitive) and psychiatric symptoms.^10^ Previous studies have sought to determine where symptoms predominantly cluster, for example (a) primary cognitive syndromes where the cognitive deficits are the signal features, (b) psychiatric syndromes in which the psychiatric symptoms are the primary features.^10,16^

The present study adopted a similar algorithmic approach, with the additional consideration of the degree to which environmental triggers and caregiver interactions contributed to the described symptoms by analysing qualitative descriptions of symptoms recorded by researchers on the NPI-NH.

In summary, the present study sought to explore the nature of symptoms rated as psychiatric in a large randomised controlled trial, and understand the impact of applying an alternative algorithm that accounted for psychiatric, cognitive, environmental and care-related factors on overall NPI-NH scores.

## Method

### Participants

Participants (*n* = 725) were recruited from 50 care homes (mean 15 residents per care home) as part of a randomised controlled trial,^17^ we present baseline data only. Permanent residents with a formal diagnosis of dementia or a score ≥4 on the Functional Assessment Staging Test of Alzheimer's disease^18^ were recruited. Residents were ineligible if they had been formally admitted to an end-of-life care pathway or were cared for in bed. The average age was 85.7 years (range 57–102). The majority of participants were women (536; 74%) and identified as White British (702; 97%). One participant was removed because of missing data.

### Measures

The NPI-NH^7^ was completed for all participants by a staff proxy with a researcher. This measure consists of 12 subscales, for example delusions, hallucinations. For each subscale the NPI-NH includes a number of predetermined questions to identify whether specific behaviour are present such as for agitation/aggression: ‘Does the resident shout, make loud noises or swear angrily?’ For each subscale there is also an ‘other’ response (except for ‘aberrant’) where staff can provide qualitative description behaviours that do not reflect the predetermined questions. For aberrant behaviours the qualitative component simply asks raters to provide more information.

If the proxy respondent answers yes to any predetermined question or provides a description of an ‘other’ behaviour they are asked to report how frequently the behaviour(s) occur on a four-point scale (from rarely to very often), the severity of the symptoms (mild, moderate or severe) and their occupational disruptiveness on a six-point scale (not at all to very severely). In this study we analysed the qualitative descriptions of behaviours recorded in the ‘other’ category.

### Data preparation

Prior to algorithm development, for patients where no qualitative description was entered in any of the symptom categories the interview was removed. This provided a total of 347 participants, who had a qualitative description of at least one symptom (median 2; range 1–8).

### Algorithm development

Three of the authors trained in the use of the NPI (S.J.S., A.W.G., and C.S.) independently thematically coded symptoms with qualitative descriptions for one-third of the 347 participants. The independent coding was predicated on a biopsychosocial approach, as first purported by Kitwood^19^ in the enriched model of dementia, and subsequently updated to inform approaches to practice^20^ and person-centred care.^21^

**Fig. 1:**
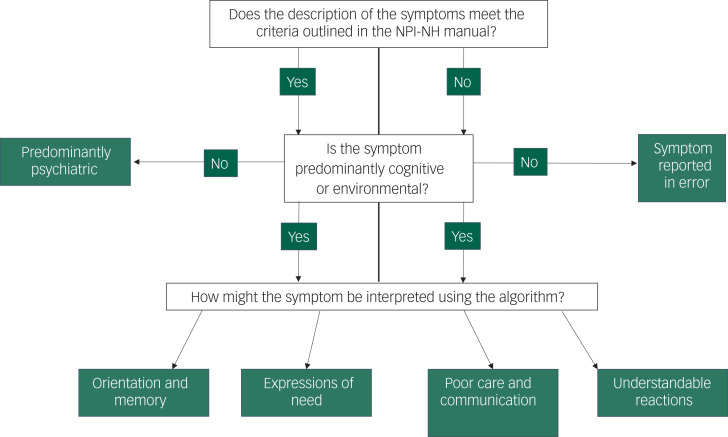
Symptom Classification Algorithm. NPI-NH, Neuropsychiatric Inventory – Nursing Home.

The process described in Fig. 1 was followed by each independent rater for each qualitative description of a symptom. The qualitative symptoms varied in length and detail. For example:

- ‘Selectively resistant’ (agitation);
- ‘Used to sing along with the radio, it is not that she has lost interest. She does not have the ability to do activities/interests any more’ (apathy);
- ‘If staff are walking past, she requires attention. Will call out and ring bell. Can be aggressive if attention not given e.g. hit staff’ (agitation).

The researchers examined the descriptions from a biopsychosocial perspective and noted where there was information that could indicate a causal interpretation of the behaviour, behavioural trigger or information that might suggest that the behaviour does not meet the threshold for being pathological or abnormal. For example ‘Doesn't like loud noise – leaves room’.

The raters then shared their interpretations of behaviours indicating causal features that could be identified in the symptom description or alternative behavioural explanations. These were reviewed across the three raters and themes were elicited that captured the potential biopsychosocial interpretations of symptoms. These themes were generated by examining how each rater had described potential causal factors, behavioural triggers or alternative interpretations that featured in the description, and generating categories based on the similarities between these features. For example, features described as negative communication, malignant social psychology or negative staff interaction were grouped into the theme ‘poor care and communication’. In the resulting algorithm there are four ways in which the symptoms can be interpreted. Where it is identified that symptoms cluster around predominantly cognitive and environmental triggers, four biopsychosocial interpretations of symptoms can be considered:

- issues with orientation and memory;
- expressions of need;
- poor care and communication; and
- understandable reactions.

The algorithm is presented in Fig. 1.

### Role of the funder and ethics

The report is based on independent research commissioned and funded by the National Institute for Health Research Health Technology Assessment programme (15/11/13). The views and opinions expressed are those of the authors and do not necessarily reflect those of the Health Technology Assessment, National Institute for Health Research, National Health Service or the Department of Health and Social Care. The authors assert that all procedures contributing to this work comply with the ethical standards of the relevant national and institutional committees on human experimentation and with the Helsinki Declaration of 1975, as revised in 2008. All procedures involving human patients were approved by NRES Committee Yorkshire and the Humber-Bradford Leeds REC (13-YH-0016). Written informed consent was obtained from all participants.

### Findings

#### Testing the algorithm for reliability

Having established the algorithm, each rater independently applied the algorithm to the data-set (347 participants and 651 qualitative symptom descriptions). The reliability of the algorithm was interrogated by establishing interrater reliability. We were interested in the consistency of rating symptoms as either:

- predominantly psychiatric symptoms;
- predominantly cognitive involving environmental triggers allocated to one of the four biopsychosocial categories (orientation and memory; expressions of need; poor care and communication; and understandable reactions); or
- being coded in error (six codes in total).

Agreement between the raters was calculated in three ways for each subscale on the NPI-NH: the percentage agreement of categorisation of symptoms between raters, Krippendorf's alpha (*k* alpha) and the mean kappa (κ) agreement between the rating pairs for example:

(*k*RaterA&B + *k*RaterA&C + *k*RaterB&C)/3

(see Supplementary Table 1 available at https://doi.org/10.1192/bjo.2020.113). Good percentage agreement was greater than 75%. Moderate κ agreement >0.40, good agreement is greater than >0.60. Scores >0.57 are considered to represent good agreement using the Krippendorf calculation.^22^

Overall, none of the subscales represented poor agreement across all agreement outcomes, although they were subject to variations across the methods. The subscale that demonstrated weakest agreement using the Krippendorf and kappa statistic was elation. This is likely because of the very few (*n* = 10) qualitatively described instances of this behaviour; percentage agreement was generally good. Conversely, agitation and anxiety demonstrated weaker percentage agreement but good agreement using Krippendorf and kappa statistics, likely because of the greater number of instances and thus variability.

Overall, there were only seven instances in which at least two raters were not in agreement regarding the symptom description. The findings indicate that overall the framework used is a reliable indicator for the qualitatively described symptoms.

#### Applying the algorithm to the data-set

The algorithm was applied to all 651 qualitatively described symptoms in the data-set. Disagreement between raters regarding the symptom attribution was resolved by consensus agreement. If at least two raters were not in agreement a discussion between the raters informed the final categorisation. The number of symptoms associated within each primary coding category is presented in Table 1.

**Table 1:** Overview of qualitative symptom classifications using the Neuropsychiatric Inventory – Nursing Home (NPI-NH) framework

Subscale	Error, *n* (%)	Predominantly psychiatric, *n* (%)	NPI: CEC, *n* (%)	CEC, *n* (%)
Delusions	3 (4)	7 (10)	57 (83)	2 (3)
Hallucinations	0 (0)	10 (53)	2 (11)	7 (37)
Agitation	2 (2)	5 (6)	52 (64)	22 (27)
Depression	4 (6)	22 (33)	22 (33)	19 (28)
Anxiety	3 (4)	21 (25)	48 (58)	11 (13)
Elation	0 (0)	0 (0)	5 (50)	5 (50)
Apathy	0 (0)	2 (5)	22 (52)	18 (43)
Disinhibition	4 (12)	6 (18)	16 (48)	7 (21)
Irritability	0 (0)	7 (12)	44 (76)	7 (12)
Aberrant	2 (2)	17 (21)	56 (68)	7 (9)
Sleep	2 (4)	0 (0)	45 (92)	2 (4)
Appetite	1 (2)	4 (7)	42 (72)	11 (19)
Total	21 (3)	101 (16)	411 (63)	118 (18)

CEC, not recognised as psychiatric symptom according to the NPI and predominantly cognitive involving environmental triggers or care interactions; NPI: CEC, classified correctly according to the instructions of the NPI but predominantly cognitive involving environmental triggers or care interactions.

Overall, most (79%) of the qualitatively described symptoms were correctly assigned as psychiatric symptoms based on the NPI-NH manual descriptors. However, when considering biopsychosocial explanations for the behaviour, 63% of these behaviours were predominantly attributed to other causes, and only 16% were coded as predominantly psychiatric. Of the remaining 21% of symptoms, 18% represented symptoms predominantly attributable to other causes (predominantly cognitive involving environmental triggers or care interactions (CEC)) that should not have been assigned as psychiatric symptoms based on the NPI-NH manual. An example from the depression category is ‘Upset when family don't visit’ that was understood using the algorithm as an understandable reaction. Finally, 3% of symptoms were recorded in error and did not represent behaviours relevant to the subscale. For example, ‘Aggression’ (depression).

Patterns of classification were relatively consistent across the subscales. The highest proportions of items assigned correctly as psychiatric symptoms but under the algorithm attributable to predominately cognitive or environmental triggers (NPI CEC) was in the sleep and delusions subscales. The subscale of apathy appeared to be the least understood, with 43% of symptoms being incorrectly assigned as psychiatric symptoms (CEC). For example, ‘When he is tired he will sleep, not do new things.’ The symptoms classified as predominately cognitive involving environmental cues or care interactions (NPI CEC and CEC) were further examined under the four biopsychosocial categories (see Table 2).

**Table 2:** Classification qualitatively described ‘other’ symptoms defined as predominantly cognitive involving environmental triggers or care interactions (CEC)

Subscale	Orientation and memory, *n* (%)	Poor care and communication, *n* (%)	Expression of need, *n* (%)	Understandable reaction, *n* (%)	Total CEC symptoms, *n*
Delusions	50 (85)	8 (14)	1 (2)	0 (0)	59
Hallucinations	5 (55)	1 (11)	3 (33)	0 (0)	9
Agitation	0 (0)	40 (54)	32 (43)	2 (3)	74
Depression	3 (7)	0 (0)	23 (56)	15 (37)	41
Anxiety	9 (15)	15 (25)	27 (46)	8 (14)	59
Elation	1 (10)	1 (10)	8 (80)	0 (0)	10
Apathy	2 (5)	2 (5)	35 (85)	2 (5)	41
Disinhibition	0 (0)	4 (17)	19 (83)	0 (0)	23
Irritability	1 (2)	13 (25)	33 (65)	4 (8)	51
Aberrant	4 (6)	1 (2)	57 (90)	1 (2)	63
Sleep	16 (34)	1 (2)	30 (64)	0 (0)	47
Appetite	9 (17)	2 (4)	42 (79)	0 (0)	53
Total	100 (19)	88 (17)	310 (58)	32 (6)	530

CEC, predominantly cognitive involving environmental triggers or care interactions.

Overall, the majority (58%) of symptoms examined within these categories (NPI CEC or CEC) were attributable to expressions of need. In the aberrant behaviour category 90% of the symptom descriptions related to expressions of need. For example, ‘shakes hands and squeezes hands’ and ‘going to the toilet excessively and becoming fidgety’. With the exception of the subcategories delusions, hallucinations and agitation, expression of need was the most common code applied to the qualitatively described symptoms.

In the category of delusions the most frequent attribution (50/59) for the symptom described was problems with orientation and memory. Behaviours described in this category were associated with problems with recognition memory, long-term memory or orientation. For example, ‘believes family members are in the building and she needs to find them’ and ‘thinks she needs to go home to see her husband and children’. In the subcategory of hallucinations five of the nine symptoms were coded as problems with orientation and memory (for example looks in mirror/sees own reflection but talks as if it is someone else).

The majority of symptoms that were not predominantly psychiatric in the subscale of agitation were related to poor care and communication (40 instances). For example, ‘can be physically aggressive, particularly if her frame is taken away. She grabs/snatched at things’. Many of these described behaviours occurred during personal care. For example, ‘fearfulness, can freeze and go rigid and it makes personal care difficult’. However, a high number of symptoms in this category (32 instances) were expressions of need. For example, ‘if staff are walking past, she requires attention’, ‘will call out and ring bell’ and ‘can be aggressive if attention not given e.g. hit staff’.

Many instances of depression were assigned correctly as psychiatric according to the manual but with a biopsychosocial lens represented predominantly cognitive features involving environmental cues or care interactions (CEC). These were attributed to either an expression of need (for example ‘crying sometimes in relation to pain’ and ‘waking during the night’) or understandable reactions (such as ‘wants to go home. Misses daughter. Quiet and sleepy’ and ‘upset when family don't visit’).

Overall, there were very few instances of elation; according to the algorithm the majority represented expressions of need (for example ‘tends to hug carers arms during these periods’ and ‘hugging and kissing’). Similarly, the majority of disinhibition behaviours were coded as expressions of need. For example, ‘very in the moment – takes clothes off if wet or uncomfortable’ and ‘will take food from other residents, will pick at himself in public areas if defecated’.

### Overall NPI scores

To understand the impact of applying the algorithm on the total NPI score we compared overall standard NPI-NH scores of the 725 participants with their scores with the qualitatively described symptoms removed. As described, total NPI scores are derived from the frequency × severity scores in each subcategory. Removing the influence of the qualitatively described symptoms means that the frequency × severity ratings are not reported when they are derived solely from the qualitatively described symptom. *t*-tests were conducted to account for the impact of scores that were predicated solely on the qualitatively described symptoms (see Table 3). The inclusion of qualitatively described symptoms described in the ‘other’ category had a significant impact on the overall NPI score (*t* = 6.14 d.f. = 24 *P* < 0.01). The NPI score indicates a higher degree of severity when the qualitatively described symptoms are included; the subcategories of delusions, anxiety, depression and irritability contribute to this effect.

**Table 3:** Total Neuropsychiatric Inventory – Nursing Home (NPI-NH) scores (frequency × severity) for each subscale with and without the inclusion of scores derived solely from qualitatively described symptoms

Subcategory and subscale	Frequency × severity score
Delusions*	
Standard score	0.88
Excluding qualitative	0.76
Hallucinations	
Standard score	0.58
Excluding qualitative	0.58
Agitation	
Standard score	2.2317
Excluding qualitative	2.2290
Depression	
Standard score	1.1393
Excluding qualitative	1.1214
Anxiety	
Standard score	1.0772
Excluding qualitative	0.9917
Elation	
Standard score	0.3214
Excluding qualitative	0.3172
Apathy	
Standard score	1.5986
Excluding qualitative	1.5945
Disinhibition	
Standard score	0.69
Excluding qualitative	0.67
Irritability	
Standard score	1.76
Excluding qualitative	1.73
Aberrant	
Standard score	1.8441
Excluding qualitative	1.8055
Total*	
Standard score	12.1159
Excluding qualitative	11.7834

* Significant group difference using *t*-test at *P* < 0.01.

## Discussion

### Main findings

Our findings suggest that the majority of qualitatively described symptoms in the NPI may relate to symptoms that are predominantly cognitive involving environmental triggers or care interactions. This raises questions about how the NPI is, or should be, used the context of informing individualised care and evaluating care practices. In the context that the NPI was designed, a medicalised explanation was attributed to all behaviours labelled as symptoms. Our findings suggest that the NPI overestimates the presence of predominantly psychiatric symptoms. Removing qualitatively described symptoms in our sample caused significant reductions in overall NPI score.

In our findings around 60% of the symptoms were attributed correctly according to the manualised instructions of the NPI-NH, which does not require raters to account for the causes of the behaviours. However, around 22% of the symptoms were reported as psychiatric symptoms in error; i.e. contrary to the NPI-NH manual, suggesting issues with user administration. Of the 651 symptoms we applied the biopsychosocial algorithm to only 16% were coded as predominantly psychiatric.

### Interpretation of our findings and comparison with other studies

The findings are in line with previous suggestions that the NPI-NH is limited by failing to take account of the other causes or explanations for behaviours.^5,23^ It is important to understand causes for behaviour in order to guide treatments and interventions. Our suggestion is the NPI-NH in its current form may over medicalise symptoms by suggesting that they are predominantly psychiatric, when symptoms may represent understandable reactions to care interactions or environmental cues that are modifiable. This has significant clinical implications in cases in which the NPI is used to guide treatment decisions, i.e. unnecessary psychiatric prescriptions.

In line with Zuidema et al,^15^ who found NPI-NH-rated apathy to be lower in environments where more activities are provided, our findings also suggest that symptoms can reflect the physical or social environment. In turn these may represent proxy indicators of poor care or less enriched care environments. Across all categories, symptoms were most commonly attributed to being expressions of need. In the context of a person-centred model of behaviour, expressions of need tend to occur in the absence of good person-centred care.^24^ For agitation, the majority of symptoms reflected poor care or communication; for example ‘can be physically aggressive, particularly if her frame is taken away’. In this instance the cue (removal of walking aid) has an impact on sense of safety/comfort, or may restrict independence. In the context of a biopsychosocial approach this behaviour may be reduced by reassuring the individual that the walking aid is nearby, or not removing the aid in the first instance.

Although behaviours were commonly seen as expressions of need, they were attributable to different causes at different rates across the subcategories. An example of this was observed in the subcategory of delusions, in which the majority of the symptoms described could be attributed to difficulties with orientation and memory, a common dementia symptomology for example ‘thinks she needs to go home to see her husband and children’. This symptom can be understood in the context of the patient experiencing anosognosia (unawareness), which results from the long-term memory deficit common to Alzheimer's disease related to hippocampal pathology.^25^ According to Morris’ model of anosognosia^26^ the experience results from the failure of the individuals’ ability to update their personal memory store. This includes personal semantic and episodic information, such as where the person is now living, meaning the person thinks they still live in the place they previously called home. Thus, the individual is unaware that they are currently living in a care home. Amendments to the wording and administration guidelines of the NPI-NH could be implemented to ensure that predominantly cognitive and non-cognitive symptoms are not conflated.

Likewise some NPI-NH subscales such as agitation/aggression include predetermined questions that describe predominantly environmentally triggered behaviours, and therefore potentially encourage raters to see all agitated behaviours as predominantly psychiatric. For example ‘Does the resident get upset when people are trying to care for him/her or resist activities such as bathing or changing clothing?’ Revising NPI-NH wording to ensure that the wording does not promote recording behaviours that are likely to have social or environmental causes, or to ensure that the predominant cause of the behaviour is recorded, is recommended.

### Implications

Our findings did suggest an element of user error. Previous findings have also suggested adaptions to the NPI-NH may improve its reliability by making it more accessible to care staff, such as adopting a diarised method with greater scope to record behavioural antecedents.^27^ Our findings would additionally recommend that users of the NPI-NH tool have a good understanding of biopsychosocial approaches to care in order to distinguish between triggers or alternative attributions for behaviour. The findings of this study also suggest that the NPI-NH might be reviewed to recognise alternative interpretations and causes of behaviours. This may require further research, development and validation with consideration of NPI-NH training, instruction manual, and administration and recording procedures.

In summary, this study has investigated the nature of qualitative descriptions of psychiatric symptoms in the NPI-NH and the degree to which these behaviours may or may not represent predominantly psychiatric symptoms. Our findings suggest that a significant proportion of symptoms may be predominantly cognitively rooted and/or environmentally triggered. It may be feasible and useful for amendments to be made to the NPI-NH that distinguish between causes of symptoms, and additional consideration be given to these factors in NPI administration and training. This would result in greater accuracy in recording predominately psychiatric symptoms in dementia and would align to best practice recommendations with regards to informing person-centred non-pharmacological treatment options as first-line treatments.

## Data Availability

The data collected for this study can be made available by contacting the corresponding author.

## Supplementary material

For supplementary material accompanying this paper visit http://dx.doi.org/10.1192/bjo.2020.113. click here to view supplementary material
