# Supplementary material for: A biopsychosocial interpretation of the Neuropsychiatric Inventory – Nursing Home assessment: reconceptualising psychiatric symptom attributions
Source: BJPsych Open. 2020 Nov 6;6(6):e137. doi: 10.1192/bjo.2020.113 (PMC7745231; doi:10.1192/bjo.2020.113)
Supplement: Supplementary file 1 [file bjosup.zip › S2056472420001131sup002.docx]

Appendix A

Inter rater agreement of the classification of symptoms based on the Framework

| **Subscale** | **Percentage Agreement** | **Krippendorf ABC** | ***K* Rater A&B** | ***K* Rater A&C** | ***K* Rater B&C** | **Mean Kappa** |
| --- | --- | --- | --- | --- | --- | --- |
| Delusions | 77% | 0·55 | 0·43 | 0·46 | 0·79 | 0·56 |
| Hallucinations | 72% | 0·57 | 0·345 | 0·345 | 1 | 0·563333 |
| Agitation | 59% | 0·53 | 0·36 | 0·37 | 0·94 | 0·556667 |
| Depression | 70% | 0·6 | 0·43 | 0·43 | 0·96 | 0·606667 |
| Anxiety | 50% | 0·56 | 0·38 | 0·37 | 0·97 | 0·573333 |
| Elation | 80% | 0·35 | 0·2 | 0·2 | 1 | 0·466667 |
| Apathy | 79% | 0·52 | 0·33 | 0·33 | 1 | 0·553333 |
| Disinhibition | 73% | 0·52 | 0·49 | 0·31 | 0·77 | 0·523333 |
| Irritability | 79% | 0·71 | 0·58 | 0·61 | 0·97 | 0·72 |
| Aberrant | 69% | 0·5 | 0·32 | 0·35 | 0·91 | 0·526667 |
| Sleep | 76% | 0·58 | 0·4 | 0·43 | 0·93 | 0·586667 |
| Appetite | 70% | 0·54 | 0·38 | 0·42 | 0·93 | 0·576667 |
